# A novel post-mortem pathogen discovery program detects an outbreak of Echovirus E7: Uganda, 2022–2023

**DOI:** 10.3389/fmicb.2025.1557576

**Published:** 2025-07-01

**Authors:** Sonja L. Weiss, Nicholas Bbosa, Gregory S. Orf, Michael G. Berg, Deogratius Ssemwanga, Sam Kalungi, Stephen Balinandi, Maximillian Mata, Henry Kyobe Bosa, Stella E. Nabirye, Joshua Buule, Tom Lutalo, Angela Havron, Robert Downing, Mary A. Rodgers, Francisco Averhoff, Gavin A. Cloherty, Pontiano Kaleebu

**Affiliations:** ^1^Abbott Pandemic Defense Coalition (APDC), Abbott Park, IL, United States; ^2^Abbott Diagnostics, Abbott Park, IL, United States; ^3^MRC/UVRI and LSHTM Uganda Research Unit, Entebbe, Uganda; ^4^Uganda Virus Research Institute, Entebbe, Uganda; ^5^Pathology Department, Mulago National Referral Hospital, Kampala, Uganda; ^6^Ministry of Health (Uganda), Kampala, Uganda; ^7^Uganda Peoples Defense Forces, Kampala, Uganda; ^8^Makerere University Lung Institute, Kampala, Uganda

**Keywords:** mortuary surveillance, metagenomics, echovirus, Uganda, enterovirus

## Abstract

**Objectives:**

Utilizing post-mortem examination for routine monitoring of infectious diseases and pandemic preparedness is a common-sense, yet uncommon, public health measure. Here, we established a novel mortuary surveillance program in Uganda that leverages the unbiased nature of metagenomic next-generation sequencing (mNGS) to detect pathogens in recently deceased individuals.

**Methods:**

Between October 2022 and December 2023, specimens and patient metadata were collected from 2,607 individuals across five mortuary sites around Kampala. Specimens were pre-screened for hemorrhagic fever viruses by RT-qPCR and a subset (*n* = 134) of RT-qPCR negatives were sequenced by mNGS.

**Results:**

A variety of DNA (herpes, parvovirus, bufavirus) and RNA (Saffold, Salivirus, HAV) viruses, vectored (*Bartonella, Rickettsia*) and nosocomial (*Enterobacter, Klebsiella*) bacterial infections, and potentially lethal respiratory pathogens (e.g., *Cryptococcus neoformans, Corynebacterium diphtheria*) were detected. A localized outbreak of Enterovirus B (EV-B), specifically a recombinant Echovirus E7, was observed in Kampala. An epidemiologic assessment indicated that most identified pathogens were acquired via direct and/or indirect contact (e.g., fecal-oral, fomites) and that other modes of transmission (e.g., food-borne, insect-vectored) played a less significant role.

**Conclusion:**

Integration of mortuary surveillance, coupled with mNGS, into public health systems represents a powerful strategy for identifying unrecognized outbreaks and monitoring the (re-) emergence of infectious diseases.

## Introduction

While zoonotic spillovers are key drivers of emergent viruses and exploring animal reservoirs for potential new zoonoses has merit (and risk) (Bernstein et al., 2022; Morse et al., 2012), one need not look any further than the mortuary to assess the most serious threats. Despite the clinical and epidemiological benefits of autopsies, there has been a worldwide decline in their use since the 1960s for a variety of reasons (Burton and Underwood, 2007; De Cock et al., 2019). These include the added time and expense being at odds with the need to rein in healthcare costs, legal aspects such as consent or fear of identifying malpractice, the belief that more advanced techniques have rendered them obsolete, and cultural proscriptions or general aversion to the procedure. Factors such as these have negatively swayed public perception against its benefits, resulting in fewer requests made by practitioners to family members for otherwise eligible deaths. Post-mortem surveillance (MS) in mortuaries, using less invasive body fluid sampling and diagnostics, is a promising alternative to autopsies. As evidenced by its successful use in several low-and middle-income countries (LMICs) to assess excess mortality associated with HIV and COVID-19 (Sheppard et al., 2023), MS can fill important gaps in countries where detailed vital statistics are lacking. As the number of outbreaks has accelerated, and technology has improved with the advent of next-generation sequencing (NGS), MS with sequence-agnostic tools could facilitate early detection of emerging/re-emerging infectious diseases.

In November of 2020, Uganda established the Uganda Rapid Mortality Surveillance Project to assess the impact of COVID-19 and excess mortality attributed to the pandemic (Uganda National Institute of Public Health, 2024). The program ended in 2021, but by September 2022 there were renewed calls for MS in response to the 2022 Ebola Virus outbreak in Uganda (Aceng et al., 2023). A dedicated program was initiated by the Uganda Virus Research Institute (UVRI) in Entebbe working together with the Ministry of Health (MoH) to conduct screening of post-mortem specimens from individuals suggestive of an infectious etiology. The Abbott Pandemic Defense Coalition (APDC) expanded the program to employ NGS to broadly surveil for circulating infectious diseases otherwise not targeted by conventional diagnostics.

We describe key findings of the Uganda MS program, including screening for VHF viruses and sequencing of select specimens, during October 2022–December 2023, as the 2022 Ebola outbreak was waning. Results revealed a variety of infections and modes of transmission, most notably the detection of an echovirus concentrated in Kampala.

## Materials and methods

### Ethical approval

The study was approved by the UVRI Research Ethics Committee (REC) (ref GC/127/908) and the Uganda National Council of Science and Technology (Reference HS2543ES).

### Study design

Five health facilities in Kampala: Mulago National Referral Hospital (MNRH) Mortuary, Kiruddu Referral Hospital Mortuary, Kampala City Council Mortuary, Naguru General Hospital Mortuary and the Uganda Cancer Institute Mortuary were selected for conducting MS for viral hemorrhagic fever (VHF) beginning 31 October 2022. Sample collection focused on individuals whose deaths were non-traumatic, unexpected, and/or had inconclusive postmortem findings of a potentially infectious etiology. Those with signs and symptoms consistent with infectious etiologies, particularly VHF, were preferentially targeted for enrollment. Collection usually occurred within 24 h of death. Two sample types were obtained: a whole blood specimen and/or an oral swab in Viral Transport media (VTM) to increase the chance of nucleic acid harvest. Whole blood specimens were also processed to isolate plasma. Specimens were collected daily from all participating mortuary sites and transported to the MNRH Pathology department before shipping them to the UVRI laboratories for processing, mNGS, or longer-term storage.

### Metadata collection

Research assistants collected data about the deceased from next of kin or medical records and recorded them on a standardized Case Investigation Form (CIF). The CIF included demographic information, clinical signs and symptoms, hospitalization information, risk factors and exposures, and the clinical specimens collected. All data was entered into an electronic case report form (eCRF).

### Pre-screening for viral hemorrhagic fevers (VHF)

Specimens were initially screened by RT-qPCR assays sourced from United States Centers for Disease Control and Prevention (US CDC) for VHF species at UVRI. RNA was extracted from individual patient specimens (no pooling) using the MagMax kit (applied Biosystems Inc., Vilnius, Lithuania) following the manufacturer’s instructions. Assays included Ebola (Balinandi et al., 2023; Nyakarahuka et al., 2022; Towner et al., 2008), Marburg (Nyakarahuka et al., 2019), Rift Valley fever (RVF) (Shoemaker et al., 2019), and Crimean Congo fever (CCHF) (Balinandi et al., 2024). Sequences of primers and probes are found in respective references.

### Next-generation sequencing of patient specimens

Plasma and swab samples were pre-treated with benzonase nuclease prior to total nucleic acid (TNA) extraction on a KingFisher Apex instrument (ThermoFisher, Waltham, MA, USA). Plasma and swab samples were extracted at a 400 μL volume (40 μL 10x Benzonase buffer, 1 μL Benzonase, 360 μL plasma or swab eluate). Volume of whole blood samples was limited thus a 200 μL volume was used for extraction for all whole blood specimens without benzonase treatment. TNA was eluted in a 50 μL volume for all specimens and stored at −80°C.

Unbiased library preparation and viral enrichment using the Comprehensive Viral Research Panel (Twist Biosciences, South San Francisco, USA) were carried out at Abbott Laboratories, Abbott Park, IL, USA, essentially as described previously (Orf et al., 2024). The mass of each NGS libraries was measured using a QubitFlex Fluorometer (ThermoFisher, Waltham, MA, USA) and library size assessed using a TapeStation 2200 Bioanalyzer (Agilent Technologies, Santa Clara, CA, USA), respectively. Libraries were diluted to 2.2 nM before equimolar pooling and final dilution to 650 pM for loading. Sequencing of virally enriched and metagenomic libraries was performed at Abbott Laboratories on an Illumina NextSeq 1000 instrument. A portion of the metagenomic sequencing was carried out at Azenta US, Inc. on a NovaSeq instrument.

### Bioinformatic analysis and genome assembly

FASTQ read files were uploaded to Abbott’s proprietary DiVir 3 bioinformatics pipeline, which utilizes BLAST (Altschul et al., 1990), BWA (Li and Durbin, 2009), MMSeqs2 (Steinegger and Söding, 2017), and SPAdes (Bankevich et al., 2012), respectively, to trim adapters, remove low quality reads, taxonomically bin, then assemble contigs. Reads not classified as background human, prokaryotic, fungal, plant, invertebrate, or known viral are translated and compared to viral protein databases using a combination of BLASTp (Altschul et al., 1990), PSI-BLAST (Altschul et al., 1997), and RAPSearch2 (Zhao et al., 2012) to assess the presence of divergent viral species.

FASTQ read files were imported into the CLC Genomics Workbench v.21 (Qiagen, Hilden, Germany) to be mapped to appropriate viral references as indicated by DiVir 3. Iterative read mapping, contig extension, and *de novo* assembly were used to complete genomes as needed. Mapping statistics were calculated and collected using CLC Genomics Workbench.

### Phylogenetic and recombination analysis of Enterovirus B genomes

Seven highly related (>99% nucleotide identity) Enterovirus B genomes were recovered at varying levels of coverage. For phylogenetic analysis, a single consensus full genome was considered. To create a dataset for maximum likelihood (ML) phylogeny, all nucleotide sequences under Taxonomy ID 138949 (species “Enterovirus B”) were downloaded from GenBank on 27 June 2024. The alignment was reduced to 1,766 non-redundant genomes and the ML method was implemented in IQTREE v.2.1.3 (Minh et al., 2020) (Supplementary information). The VP1 and 3D regions were also extracted from the full genome alignment and separately analyzed in IQTREE.

To assess recombination, the consensus Enterovirus B genome was divided every 500 nucleotides, and each fragment was individually subjected to BLAST against a viral *nt* database to recover the top 100 hits. The full genomes for each set of 100 hits were downloaded, combined into a single dataset, and redundant genomes were discarded. We then followed a previous protocol (Pikuła et al., 2021; Perez et al., 2023) using the Recombination Detection Program 5 (RDP5) (Martin et al., 2021) with seven methods: RDP, GENECOV, Bootscan, MaxChi, Chimera, SisScan, and 3SEQ. Recombination was considered only when all methods detected recombination signal, with scores below a *p* value of 0.01 when Bonferroni’s correction was applied.

### Statistical analysis of case metadata

Case metadata were analyzed using Stata v.17 (Stata Corporation, College Station, USA), and *R* v.4.3.1 with packages *tidyverse*, *lubridate*, and *zoo*. Geographic data were downloaded in vector format from a public domain source.^1^

## Results

### Implementation of MS program in Uganda

In September 2022, the Uganda MoH declared an Ebola virus outbreak in Mubende district and instituted response measures. As Ebola continued to spread beyond Mubende to neighbouring districts like Kassanda, a 3-week lockdown in Mubende and Kassanda was imposed in October 2022, with a directive issued to screen all deaths for Ebola and other VHFs. The MS study described here began in late October 2022 at the conclusion of the Ebola outbreak.

#### Patient recruitment, specimen collection, screening, sequencing, and reporting

**Figure 1:**
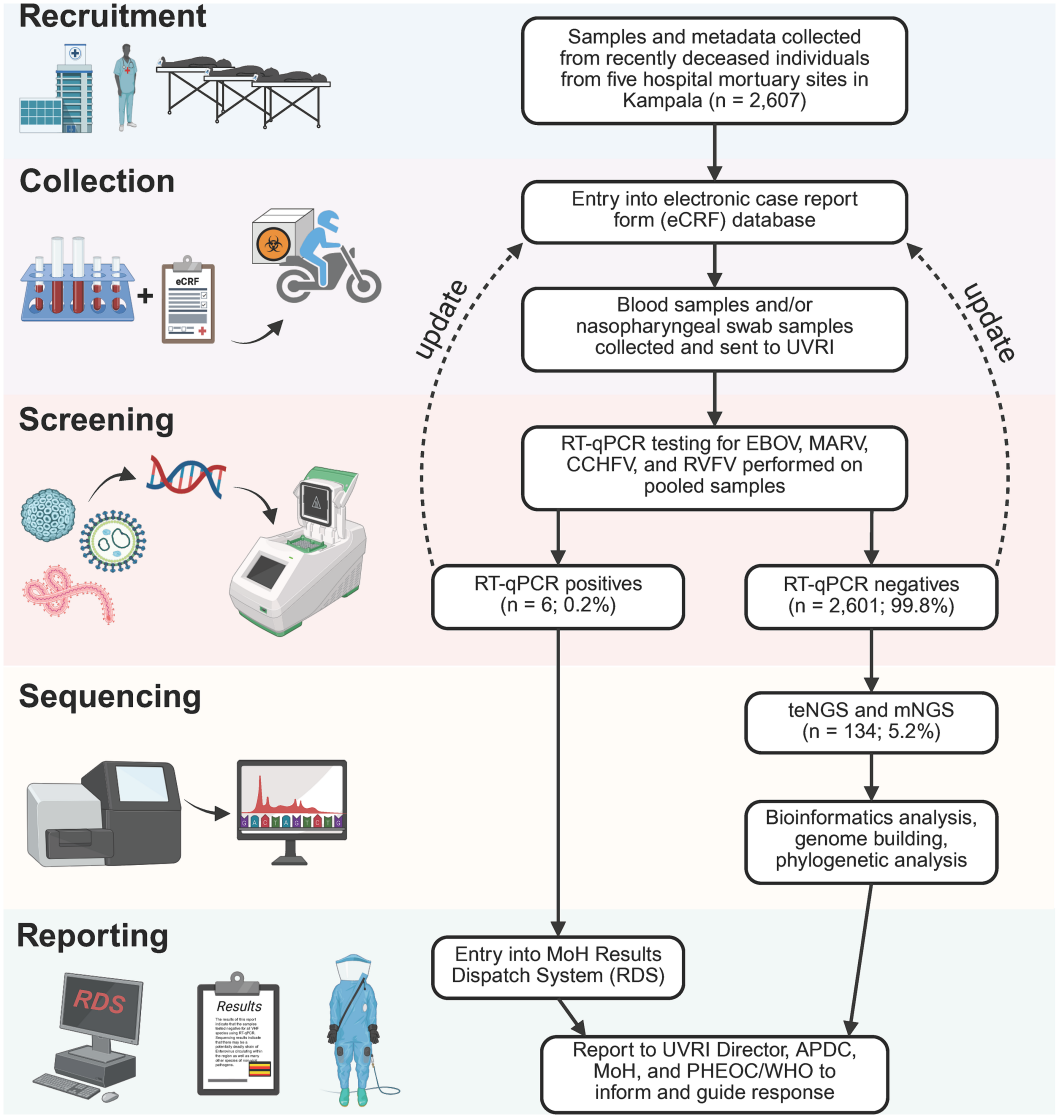
Mortuary surveillance study design. 2,607 specimens were collected from deceased individuals arriving at 5 mortuaries in Kampala from 31 October 2022 to 12 December 2023. Specimen types included blood and/or nasopharyngeal/oral swabs–specimen aliquots first sent to the National VHF Reference laboratory to screen for Ebola, Marburg, CCHF, and RVF viruses. Positive PCR results for any of the VHF viruses is reported in the Uganda MoH Results Dispatch System (RDS) to inform response as well as to the UVRI Director, Public Health Emergency Operations Center (PHEOC) and the APDC. Specimens identified as PCR negative proceed to the metagenomics sequencing pipeline to detect known or novel pathogens of pandemic potential. A total of 134 VHF-negative samples were selected for mNGS, from October 2022 to February 2023. Fastq deep sequence files generated from the mNGS were analyzed using the DiVir3 bioinformatics analysis pipeline.

**Figure 2:**
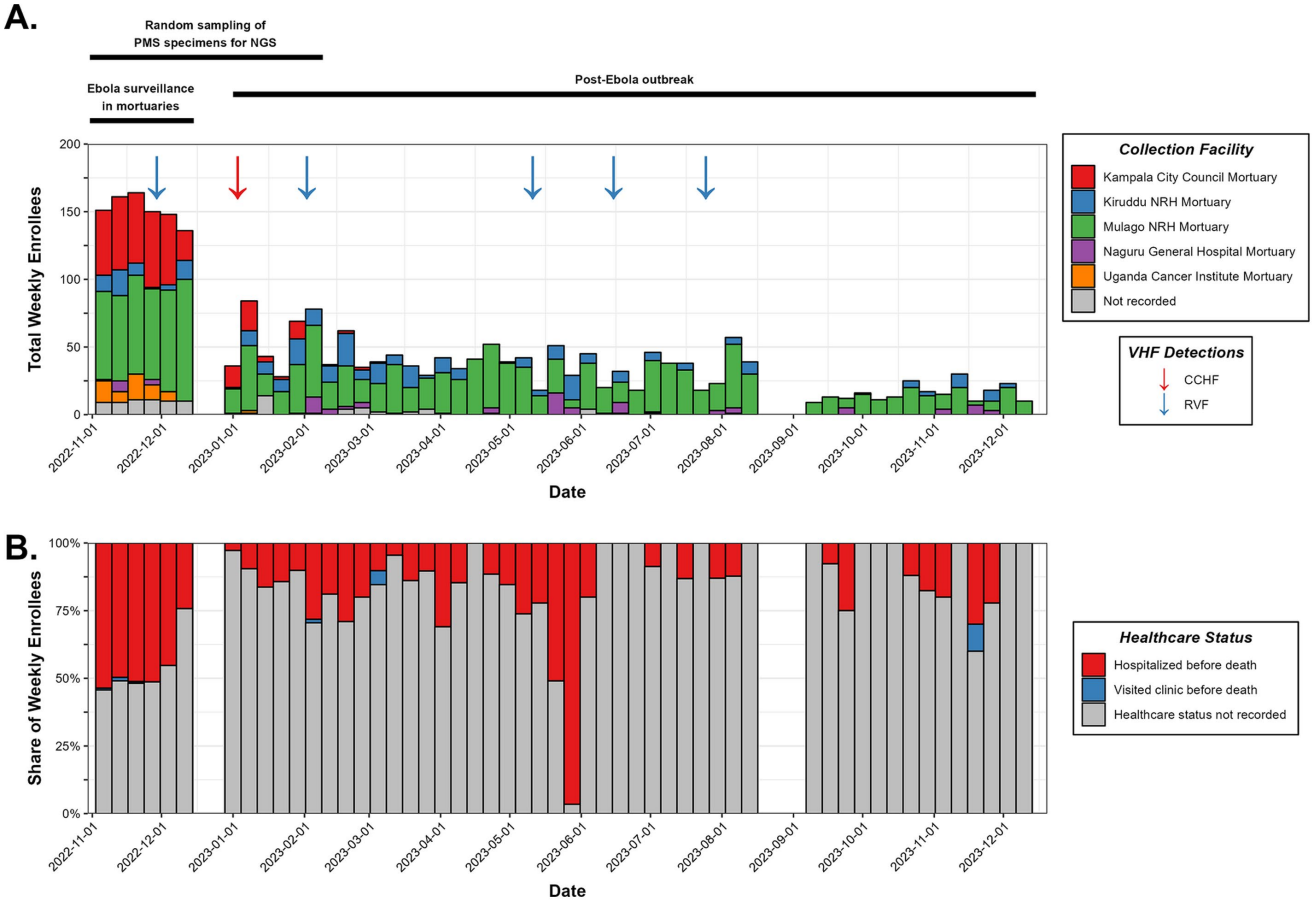
Specimen collection characteristics. **(A)** Accounting of the individuals enrolled in mortuary surveillance during the study period, summarized weekly with collection facility and detected VHF cases denoted. A timeline of the surveillance program’s major events is shown at the top. **(B)** Healthcare status of deceased individuals prior to death.

Metadata and specimens from deceased individuals in Kampala were collected, transported, and processed as illustrated in Figure 1 and described in Materials and Methods. Results of VHF screening and NGS analysis were shared with the Ministry of Health to inform public health responses. During the study period, October 2022–December 2023, 2,607 deceased individuals were enrolled. Among the 35.7% with age recorded, age >60 years (11.4%) was the most reported (Table 1). The majority (53.7%) were males (Table 1). Most cases (1,623; 62.3%) were recruited from Mulago Hospital, the largest referral hospital in the country (Figure 2A). Six (0.3%) of the 2,607 screened tested positive for a VHF including five cases of RVF and one of CCHF (Figure 2A). Of the total 2,607 decedents enrolled during the study period, 1,381 were enrolled during the Ebola outbreak and the 6 weeks following its conclusion. From this period, 134 (9.7%; or, 5.2% of all 2,607 enrolled) individuals with comparatively more complete metadata and increased numbers of symptoms were selected for sequencing at Abbott (Figure 2A; Table 1, Supplementary Table S1). Epidemiological curves enumerating enrollment (Figure 2A), and the percentage of participants who were hospitalized or visited a clinic before death (Figure 2B), were compiled based on available information.

**Table 1:** Demographic and clinical sign and symptom data collected.

	Category	*n* (% all)	*n* (% of NGS cohort)
Characteristic			
Age (years)	<18	83 (3.2)	16 (11.9)
	18–29	167 (6.4)	7 (5.2)
	30–39	141 (5.4)	13 (9.7)
	40–49	128 (4.9)	15 (11.2)
	50–59	115 (4.4)	10 (7.5)
	>60	296 (11.4)	16 (11.9)
	Not reported	1,677 (64.3)	57 (42.5)
Sex	Male	1,399 (53.7)	80 (59.7)
	Female	1,171 (44.9)	52 (38.8)
	Not reported	37 (1.4)	2 (1.5)
District of illness	Kampala	405 (15.5)	27 (20.1)
	Luwero	34 (1.3)	3 (2.2)
	Mukono	81 (3.1)	7 (5.2)
	Wakiso	233 (8.9)	20 (14.9)
	Other	162 (6.2)	19 (14.2)
	Not reported	1,692 (64.9)	58 (43.3)
District of death	Kampala	501 (19.2)	31 (23.1)
	Luwero	47 (1.8)	4 (3)
	Mpigi	35 (1.3)	4 (3)
	Mukono	89 (3.4)	7 (5.2)
	Wakiso	332 (12.7)	26 (19.4)
	Other	297 (11.4)	27 (20.1)
	Not reported	1,306 (50.1)	35 (26.1)
Occupation	Business/trade	128 (4.9)	6 (4.5)
	Healthcare worker	6 (0.2)	1 (0.7)
	Farmer	181 (6.9)	17 (12.7)
	Animal worker	8 (0.3)	2 (1.5)
	Transporter	15 (0.6)	8 (6.0)
	Not employed outside the home	141 (5.4)	8 (6.0)
	Student/child	60 (2.3)	10 (7.5)
	Government/civil	30 (1.2)	1 (0.7)
	Other	4 (0.2)	2 (1.5)
	Not reported	2,023 (77.6)	79 (59.0)
Clinical signs and symptoms			
Fever signs	Yes	59 (2.3)	37 (27.6)
	No	3 (0.1)	0 (0)
	Not reported	2,545 (97.6)	97 (72.4)
Hemorrhagic symptoms	Yes	33 (1.3)	17 (12.7)
	No	6 (0.2)	10 (7.5)
	Not reported	2,568 (98.5)	107 (79.9)
Respiratory/cardiac symptoms	Yes	384 (14.7)	55 (41.0)
	No	2 (0.08)	0 (0)
	Not reported	2,221 (85.2)	79 (59.0)
Gastrointestinal symptoms	Yes	302 (11.6)	48 (35.8)
	No	4 (0.2)	2 (1.5)
	Not reported	2,301 (88.3)	84 (62.7)
Constitutional/CNS/other	Yes	457 (17.5)	59 (44.0)
	No	0 (0)	0 (0)
	Not reported	2,150 (82.5)	75 (56.0)

Column 3 reports the results of all patients in the cohort (*n* = 2,607) and column 4 shows only those sequenced (*n* = 134).

### Diverse viral infections were detected including an Echovirus E7 outbreak in the Kampala/Wakiso vicinity

**Figure 3:**
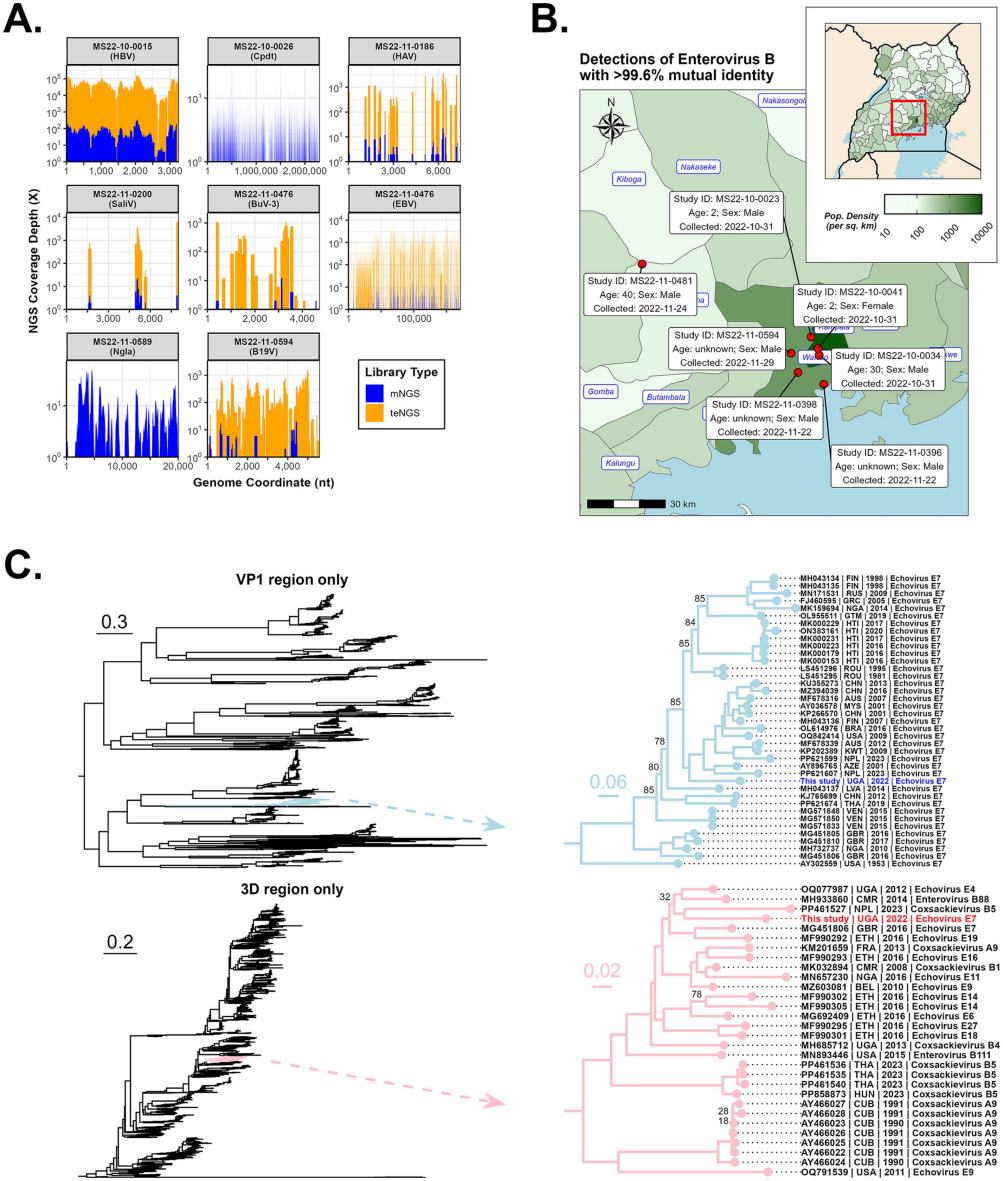
Detection of various pathogens by NGS, including an outbreak of Echovirus E7. **(A)** Genome coverage of selected pathogens processed by both mNGS and teNGS. **(B)** Geographical distribution of the detected recombinant Enterovirus B cases (i.e., place where the patient lived or became ill) with relevant case metadata (e.g., collection date). **(C)** Maximum likelihood phylogeny was performed on VP1 (top) and 3D (bottom) regions extracted from alignments of 1,766 complete Enterovirus B genomes. Branches containing the Kampala strain are shown. Ultrafast Boostrapping support values are shown only at those nodes where support is below 90%.

Whole blood and swabs specimens (*n* = 134) were converted to mNGS and teNGS libraries and sequenced independently. Target enrichment increased sensitivity to enable detection of a variety of virus families which were confirmed by both methods (Figure 3A).

#### HxV

Blood-borne viruses were detected in six people: HBV (*n* = 2), HCV (*n* = 1), and HIV (*n* = 4) (Figure 3A). Subject UVRI-MS-0112 was co-infected with HBV (genotype 4 V) and HCV (genotype D7). The other HBV sequence from UVRI-MS-001 was Genotype A. Partial (9–89%) HIV-1 sequences were constructed for 4 individuals and grouped with subtype A (*n* = 1), subtype D (*n* = 2) and one unique recombinant form (URF) subtype AD isolated from an individual in Uganda (NCBI Accession MW006064), while the HCV sequence was genotype 4. These viruses’ role in cause of death are unclear, however, their immunosuppressed status may have increased the likelihood of contracting opportunistic infections. Indeed, fungal species *Nakaseomyces glabratus* and *Cryptococcus neoformans* were detected in HIV+ subjects UVRI-MS-0043 and UVRI-MS-0133, respectively.

#### DNA viruses

Several herpesviruses were detected: human herpesvirus-4 (Epstein–Barr, EBV; *n* = 3), human herpesvirus-6B (roseola; *n* = 1), and human herpesvirus-8 (Kaposi Sarcoma; *n* = 2) (Figure 3A). Fever, fatigue, and sore throat were common among the EBV+. Bufaviruses (BuV; *n* = 2) are members of the parvovirus family and have been associated with gastroenteric disease in humans. Partial genomes for BuV-3 and BuV-1 were assembled for UVRI-MS-0020 and UVRI-MS-0086, respectively. A potential co-infection of EBV and BuV-3 was seen in UVRI-MS-0020. Patient symptoms were consistent with this finding and included diarrhea, fatigue, loss of appetite, and abdominal pain. Parvovirus B19 (B19V) is highly infectious and common in childhood with symptoms including fever, headache, nausea, body aches, and a distinctive red rash on the cheeks. B19V was detected in three adult individuals UVRI-MS-0044, UVRI-MS-0045, and UVRI-MS-0101.

#### RNA viruses

Besides HCV, HIV, and enteroviruses (discussed below), three additional ssRNA viruses were detected, all belonging to the Picornavirus family: Hepatitis A virus (HAV), Salivirus, and Saffold Virus (SAFV) (Figure 3A). Notably, 10/134 (7.5%) of postmortem specimens detected a picornavirus by NGS. HAV is food/water-borne and generally self-limiting, causing illness for weeks to months. The lone case of HAV (UVRI-MS-0065) was in a 3-year-old female who also had numerous reads for *Plasmodium falciparum* (malaria). Consistent with HAV infection, she exhibited symptoms of fever, nausea, abdominal pain, and jaundice. Saliviruses are associated with self-limiting, acute gastroenteritis, typically in children, but complications due to severe diarrhea could prove fatal. The partial Salivirus genome assembled here was from UVRI-MS-0066, a 73-year-old female. While typically a gastrointestinal virus, patient symptoms included chest pain, cough, and difficulty breathing. SAFV is an emerging cardiovirus associated with aseptic meningitis, gastroenteric, and respiratory infections. The one-year-old child (UVRI-MS-0087) exhibited fever, difficulty breathing and swallowing, sore throat, and mental confusion.

#### Enterovirus

The most frequently detected viruses were enteroviruses (*n* = 8; *Picornaviridae* family). Enterovirus-A from UVRI-MS-0087, a one-year-old male, yielded 19% coverage and bore 95% nucleotide identity to NCBI accession MH685713 (Enterovirus E0-67) from Uganda. Symptoms included fever, difficulty breathing and swallowing, sore throat, and loss of consciousness. Enterovirus B (EV-B) was prominent (5.2%; 7/134), with BLAST hits matching to Echovirus-E4 and Echovirus-E7 serotypes. Read/million counts were high in all plasma specimens, ranging from 43,360–891,528 (CVRP) to 2–5,741 (mNGS). Of note, the Echovirus-E7 serotype has been associated with severe manifestations, including virus-associated hemophagocytic syndrome (VAHS) and even death in young children (Pudjiadi et al., 2021; Watanabe et al., 2019). The clinical presentation for one male age 2.8 years, UVRI-MS-0006, included fever*, nausea, diarrhea*, fatigue*, loss of appetite, abdominal pain*, chest pain*, muscle pain, cough*, difficulty breathing* and swallowing, sore throat*, jaundice, rash, hiccups, and loss of consciousness* before succumbing to death. Patients MS-0004 and MS-0007 exhibited many of these symptoms (indicated by *), whereas no data was available for the other 4 cases.

Notably, all seven EV-B cases were collected between 31 October 2022 and 29 November 2022. Six of the patients lived or became ill within the same 20-km radius in Wakiso/Kampala; the seventh case was from an area roughly 60 km away, in the neighboring Mityana District (Figure 3B). Enterovirus-B strains had >99.6% nucleotide identity, which combined with their spatiotemporal similarities, is indicative of an outbreak. BLASTn and BLASTp analysis of the consensus outbreak genome indicated ~84% nucleotide and ~97% protein identity, respectively, to Echovirus-E7 (e.g., accessions LS451295 and MG451806). The 1ABCD region of the virus is post-translationally proteolyzed into four structural proteins, VP1-VP4. VP proteins are the most variable regions of the genome, tend to be transferred together when recombined, and dictate host tissue tropism (do Socorro Fôro Ramos et al., 2021; Hyypiä et al., 1997; Kitamura et al., 1981; Oberste et al., 1999). The VP region is used to classify enteroviruses into serotypes and an ML tree of the VP1 region alone shows the consensus genome confidently branches with Echovirus E7 (Figure 3C ***-*** top). It is the most basal taxon within a clade containing viruses isolated over the past 20 years from Asia, South America, Europe, the Middle East, and North America. By contrast, the 3D region (RdRp) branches with a variety of strains representing diverse Echovirus serotypes E4/E7/E9/E14/E19 and Coxsackivirus serotypes B1/B4/B5 isolated in the past 20 years from Africa and Europe (Figure 3C ***-*** bottom), including an Echovirus-E4 strain (OQ077987) isolated in Uganda in 2012. Both VP1 and 3D trees indicated that GenBank accession MG451806, an Echovirus-E7 isolated from the United Kingdom in 2016, is closely related to the Kampala strain.

Recombination occurs frequently in enteroviruses due to natural processes of co-circulation and co-infection within populations (Muslin et al., 2019), and acts as a major driver for the accumulation of genetic diversity. Hotspots for recombination breakpoints tend to occur on either side of the 1ABCD region of the polyprotein, but not within (Nikolaidis et al., 2019). An ML tree of complete EV-B genomes was reconstructed (Supplementary Figure S1) and the Kampala strain was resolved into a well-supported clade containing recent Echovirus-7 sequences from Nigeria and the United Kingdom. However, branch lengths between it and its closest relatives reveal an evolutionary divergence of over 0.3 substitutions per site and suggest these sequences are not directly linked (Supplementary Figure S1). The 5′-UTR and the three major regions of the polyprotein were individually subjected to nucleotide BLAST searches and the top hits for each region were different, including Echoviruses-4/6/7/9/11/18/20, Coxsackievirus-A9/B3/B4/B5, and Enterovirus-B69/B80, indicating the consensus outbreak genome likely contains genetic elements from different Enterovirus-B serotypes (Supplementary Table S2). The recombination hypothesis was tested as described in the Supplementary information and results of seven statistical methods and *p*-values are listed (Supplementary Table S3). Recombination breakpoints were detected and are situated roughly at the beginning and end of the 1ABCD region (start: nucleotide 860, polyprotein residue 50; end: nucleotide 3,327, polyprotein residue 862) (Supplementary Table S3 and Supplementary Figure S1). The major parent of this recombination event appears to be related to the Echovirus-E4 strain identified in Uganda in 2012 (GenBank accession OQ077987) (Ashraf et al., 2023), while the minor parent appears to be related to an Echovirus-E7 strain identified in a China in 2001 (GenBank accession KP266569). In the major parent regions, the Ugandan Echovirus-E4 strain bears 85.4% identity to our Kampala strain, while in the minor parent region, the Chinese Echovirus-E7 strain bears 82.7% identity to our Kampala strain (Supplementary Table S3). RDP and Bootscan methods also detected the possible introduction of genetic information from a virus related to another Ugandan strain from 2013 (Coxsackievirus-B3; GenBank accession MH685712) in the 5′-UTR (*p*-values of 7.730 × 10^−3^ and 3.443 × 10^−3^, respectively). However, since only two methods detected this signal, this result is speculative.

### Non-viral pathogens and routes of transmission suggest multiple sources of infection

#### Bacteria

**Figure 4:**
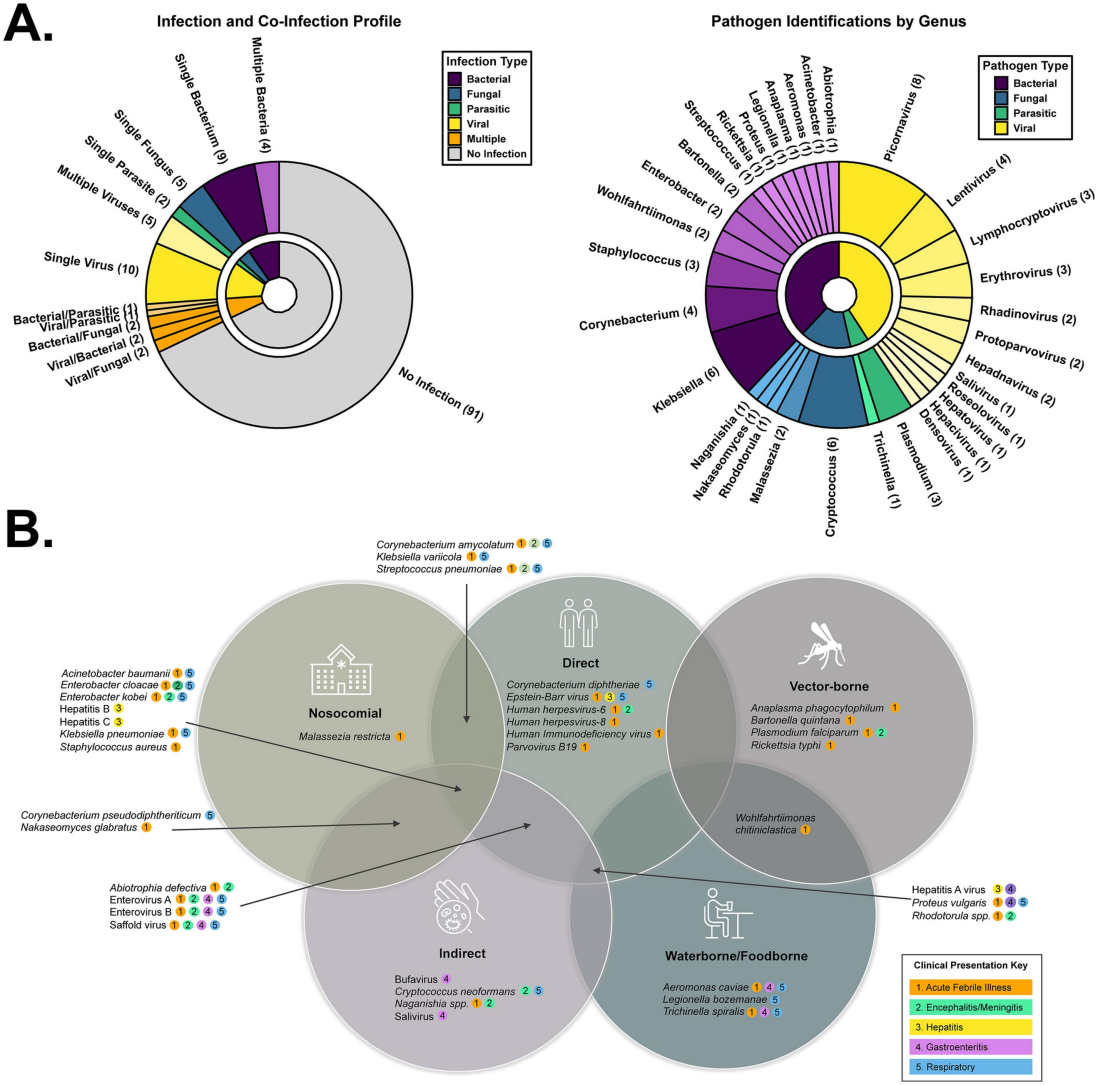
Taxonomic and transmission profile of the pathogens detected during this study. **(A)** Pie charts depicting the (co-) infection profile of the 134 sequenced specimens (left) and the genera of those pathogens (right). **(B)** Venn diagram depicting the distinct or shared transmission routes of the detected pathogens. Each pathogen also has a color key describing its various possible clinical presentations.

NGS only made determinations in 45/134 (33%) of post-mortem cases, which is perplexing since ‘resolved’ infections cease to be an explanation. Viruses made up the majority (*n* = 23), with bacteria (*n* = 17), parasites (*n* = 4) and fungi (*n* = 10) also present (Figure 4A-left) individually or as co-infections. Pathogens were the classified by genus (Figure 4A-right). We observed a potential case of trench fever, caused by the bacterium *Bartonella quintana* and transmitted by body lice. This individual showed the classic symptoms of fever, joint/muscle pain, conjunctivitis, and mental confusion/loss of consciousness. Hallmark pain in the legs (shins) and back was not reported. Additional Rickettsial species were detected, including *Anaplasma phagocytophilum* (*n* = 1) and *Rickettsia typhi* (*n* = 1). *Corynebacterium diphtheriae*, the causative agent of diphtheria, was detected in three individuals exhibiting symptoms including fatigue, chest pain, difficulty breathing, and bloody cough. Recent epidemiological data suggests this vaccine-preventable disease is on the rise in several African countries (World Health Organization, 2024).

*Klebsiella pneumonia* (*n* = 5) and *Klebsiella* var*iicola* (*n* = 3) were also frequently observed. These opportunistic, nosocomial bacteria are of increasing concern, particularly as antimicrobial resistance expands and renders these infections difficult to treat (Kivumbi and Standley, 2021). Of similar concern to the immunocompromised are *Acinetobacter baumannii*, *Aeromonas caviae, Corynebacterium amycolatum,* and *Legionella bozemanae*. *Wohlfahrtiimonas chitiniclastica* is another vectored cause of bacterial sepsis, transmitted by maggots or flies, however, it can also be contracted by ingestion of contaminated food or water. Due to an intracellular life cycle that challenges detection, absence in plasma (e.g., not sepsis), and suboptimal extraction procedures for a given species, it is likely the number of bacterial infections we report is an underestimate.

#### Parasites

*Plasmodium falciparum* reads were detected in 3/134 individuals. Death from malaria is common in African children under the age of 5. *Trichinella spiralis* (1/134) is the causative agent of trichinosis, resulting from the consumption of undercooked or wild meat. Clinical presentation of both these fatal parasitic diseases is non-specific, leading to missed or incorrect diagnoses. *Cryptococcus neoformans*, a fungal pathogen typically affecting the immunocompromised, is the most common cause of meningitis in adults living in sub-Saharan Africa due to the high prevalence of HIV. It can also cause pneumonia-like symptoms such as cough, chest pain, fever, headache, nausea, vomiting, photophobia, and mental changes. In addition to *Cryptococcus neoformans* (*n* = 6), other opportunistic fungal pathogens detected in the cohort included *Malassezia restricta* (*n* = 2), *Naganishia* spp. (*n* = 1), *Nakaseomyces glabratus* (*n* = 1), and *Rhodotorula* spp. (*n* = 1).

#### Epidemiology

We took two approaches to categorize pathogens based on their typical manifestations and their route of transmission. Manifestations include acute febrile illness, meningitis/encephalitis, hepatitis, respiratory, and gastroenteric disease (Figure 4B). Of the 46 individuals with NGS-based determinations, most had a pathogen implicated in acute febrile illness (AFI) or encephalitis (93%), followed by respiratory (56%), gastrointestinal (GI) (26%), or hepatitis (7%). Transmission categories include direct contact (person to person, droplet), indirect contact (fecal/oral route, airborne/inhalation, fomites), vector-borne, food-borne, water-borne, blood-borne, and opportunistic/nosocomial infections (Figure 4B). There were examples in every classification, with direct and indirect contact being the primary modes of infection, consistent with the higher prevalence of AFI, respiratory, and GI disease.

## Discussion

Interrogation of undiagnosed deaths by mNGS is, to our knowledge, an innovative addition to traditional forms (e.g., PCR, serology) of MS. Our objective was to detect high risk, deadly pathogens and inform appropriate public health responses. While we identified many areas for improvement, the basic framework outlined here could serve as a blueprint for other countries seeking to implement similar programs. As a collaborative effort between UVRI, the Uganda MoH, and APDC, we adopted a strategy of building capacity in NGS, bioinformatics, and diagnostic assay development to enable early detection and rapid, scalable responses to outbreaks (Averhoff et al., 2022). For LMIC where VHF are prevalent, PCR screening is built in, not only to alert public health authorities but to prevent downstream handling of dangerous samples by personnel working in lower biosafety level laboratories. Indeed, these efforts flagged sporadic cases of CCHF and RVF. In this pilot study, samples were shipped to Abbott for sequencing, however NGS capacity exists at UVRI, and subsequent specimens have been evaluated ‘in country’. By delivering cloud-based bioinformatic tools to local scientists, overall decreases to cost and turnaround time make the findings actionable. Recent suspicious deaths in Kyotera District serve as an example of how an investigation aided by mNGS holds promise for MS (Bbosa et al., 2025). Field response teams collected relevant clinical and epidemiological data involving 27 deaths of unknown etiology and NGS revealed the presence of *Bacillus anthracis*. With this knowledge, public health officials were able to inform at-risk communities to interrupt its spread.

Given that cases of the Echovirus-E7 strain occurred during the rainy season (October and November are Uganda’s second rainy season marked by heavy rains), it is possible people were indoors more often, which facilitates spread. Enteroviruses spread more easily indoors due to higher concentrations of infectious particles, especially in poorly ventilated areas, increasing the chance of inhalation and contact with fomites (La Rosa et al., 2013). Unfortunately, we did not possess an additional specimen matrix (e.g., CSF) to sequence or other definitive lab results from individuals to confirm systemic dissemination, therefore we caution that with plasma NGS data alone we cannot assign Enterovirus B as the cause of death. It is notable that several recent outbreaks of ‘mysterious illnesses’ in neighboring countries like the Democratic Republic of Congo have eventually been attributed to malaria, exacerbated by malnutrition or dehydration (Reuters, 2025). Uganda is a malaria endemic region and the extent to which these additional factors also contributed to mortality, both in those cases where NGS identified a pathogen as well as in the 68% with no result (Figure 4A), cannot be determined.

Recombination events in enterovirus species are a driver of new variants of concern and potential epidemics of severe disease (Oberste et al., 1999). The spread of this hitherto unreported strain may be underrepresented, as only 134 mortuary cases in the Kampala area were sequenced. The relatively low nucleotide identity of the constitutive regions of the recombinant versus their parents (<90%) could imply two scenarios: (1) significant genetic divergence has occurred in the recombinant (and thus some time has passed) since the recombination event occurred, or, (2) significant genetic divergence occurred since parental ancestors were sampled and the recombination event occurred relatively recently. The Kampala strain only shares 82–85% sequence identity with accessions identified as most closely related to the putative major and minor parents (Uganda-2012 and China-2001, respectively). Given the high mutation rate of enteroviruses and the temporal gap between these reference strains and the current isolate, they should not be considered direct parental strains. Also, the VP1 and 3D trees indicated that GenBank accession MG451806, an Echovirus-E7 isolated from the United Kingdom in 2016, may be a relative to the Kampala strain. It is possible that (1) it shares a similar recombination pattern that occurred independently, or (2) it has some association with the recent ancestry of our strain.

NGS uncovered several viral and bacterial infections transmitted by multiple routes (Figures 2, 4). Direct and indirect contact exposures outweighed vector or water/food borne routes. Perhaps the most concerning was diphtheria, a vaccine-preventable disease with a mortality rate reaching 30%; Uganda is currently working to increase uptake of the three-dose series vaccine (Ssebagereka et al., 2024). Nosocomial infections were also prevalent and, along with the higher cost of healthcare in urban settings, unfortunately reinforce the practice of avoiding hospitals. Note that very few individuals sought medical care prior to their death (Figure 2B), likely seeking traditional healers instead. We did not explore whether the bacterial strains we detected possessed anti-microbial resistance (AMR) markers, however *Klebsiella pneumonia* and *Staphylococcus aureus* were responsible for a combined 10,000+ AMR associated deaths in Uganda in 2019. These NGS results can help public health officials tailor interventions accordingly. Implementing mortuary surveillance with NGS is not without logistical hurdles; however, the impact it should have to guide surveillance and improve health outcomes makes it worth the effort and commitment of resources.

Indeed, our pilot study encountered many obstacles, and we openly concede its limitations. We only sequenced 134/2,607 (5%) specimens, so results are not comprehensive. These samples were from a brief timeframe following the conclusion of the Ebola outbreak and were not distributed throughout the year. Another deficiency was the incompleteness of patient metadata, including basic demographic details which impacted our ability to perform accurate case-tracing and downstream epidemiologic analyses (Supplementary Table S1). As a result of this study and analysis, efforts to improve this aspect were implemented, and preliminary results suggest simple and low-cost interventions can greatly increase the quality of the data collected. Finally, mNGS is not an approved diagnostic, nor are participating labs CLIA certified, thus we cannot report out individual results to clinicians. However, given the MS participants are deceased, restrictions could be relaxed such that the community benefits. Building and sustaining the infrastructure for mortuary surveillance with NGS as a central component should be prioritized for control of emerging and re-emerging pathogens and integrated into public health programs in sub-Saharan Africa and beyond.

## Data Availability

Full-length sequences (>90%) of Echovirus-E7 were deposited in GenBank under accessions PV563575-PV563580 and SRA BioProject PRJNA1254727. Scripts required for building figures can be made available upon request.

## Footnotes

^1^ www.naturalearthdata.com/downloads
