# Supplementary material for: A novel post-mortem pathogen discovery program detects an outbreak of Echovirus E7: Uganda, 2022–2023
Source: Front Microbiol. 2025 Jul 1;16:1557576. doi: 10.3389/fmicb.2025.1557576 (PMC12259650; doi:10.3389/fmicb.2025.1557576)
Supplement: Supplementary file 1 [file Data_Sheet_1.docx]

**Supplemental Information**

A novel post-mortem pathogen discovery program detects an outbreak of Echovirus-E7: Uganda, 2022-2023

Sonja L. Weiss^1,2^*, Nicholas Bbosa^1,3,4^*, Gregory S. Orf^1,2^, Michael G. Berg^1,2^, Deogratius Ssemwanga^3,4^, Sam Kalungi^5,6^, Stephen Balinandi^4^, Maximillian Mata^1,2^, Henry K. Bosa^6,7,8^, Stella E. Nabirye^4^, Joshua Buule^4^, Tom Lutalo^4^, Angela Havron^1,2^, Robert Downing^4^, Mary A. Rodgers^1,2^, Francisco Averhoff^1,2^, Gavin A. Cloherty^1,2^, Pontiano Kaleebu^3,4^

*Co-first author

Affiliations:

1. Abbott Pandemic Defense Coalition (APDC)
2. Abbott Diagnostics
3. MRC/UVRI & LSHTM Uganda Research Unit
4. Uganda Virus Research Institute
5. Mulago National Referral Hospital, Pathology Department
6. Ministry of Health, Uganda
7. Uganda Peoples Defense Forces
8. Makerere University Lung Institute

**Materials and Methods**

*Phylogenetic and recombination analysis of Enterovirus B genomes*

To create a dataset for the reconstruction of a maximum likelihood phylogeny, all nucleotide sequences under Taxonomy ID 138949 (species “Enterovirus B”) were first downloaded from GenBank on 27 June 2024. Sequences under 6,000 nt were discarded and any duplicate sets sequences were reduced to a single representative, resulting in 1,766 unique genome entries. Due to varying levels of coverage in the 5’ and 3’ untranslated regions across the dataset, a multiple sequence alignment was generated using the L-INS-i algorithm of MAFFT v.7.487 [1]. Also, the VP1 and 3D regions of the alignment were extracted into their own datasets. The evolutionary histories of the three datasets were reconstructed using the maximum likelihood (ML) method implemented in IQTREE v.2.1.3 [2]. First, the ModelFinder algorithm [3] was used to identify the most suitable nucleotide substitution model (here, SYM+R10) using Bayesian Information Criterion as the scoring method. The initial ML tree was calculated using a stochastic algorithm and then optimized using the Nearest Neighbor Interchange [4, 5] heuristic method. The tree with the best log-likelihood score (the ML tree) was retained and branch supports were calculated using 1,000 replicates of Ultrafast Boostrapping [6] The ML tree was rooted at the midpoint using Dendroscope v.3.8.10 [7] and visualized using the *ggtree* package [8] for the R v.4.3.1 programming language.

To assess recombination within the consensus Enterovirus B genome, a new dataset was created. The consensus genome was divided every 500 nucleotides, and each fragment was individually subjected to a BLAST search against a viral *nt* database. The genomes (>6,000 nt) corresponding to the top 100 hits for each fragment were collected from GenBank on 27 June 2024, combined, and de-duplicated and aligned (as described above), resulting in a dataset containing 343 genomes. We then followed a previous protocol [9, 10] with minor modifications. Briefly, the multiple sequence alignment was analyzed for recombination signals using Recombination Detection Program 5 (RDP5) [11] using seven methods: RDP, GENECOV, Bootscan, MaxChi, Chimera, SisScan, and 3SEQ. Default parameters were modified for linear RNA viruses as described in the user manual. Recombination scenarios involving our consensus Enterovirus B sequence of interest were considered only when all seven methods detected recombination signal, with scores below a *p* value of 0.01 when Bonferroni’s correction was applied. Scenarios were confirmed when well-supported by bootstrap values >75% by Bootscan analysis and *p* values of <0.001 by the Shimodaira-Hasegawa tree topology test.

*Statistical analysis of case metadata*

Case metadata were analyzed using Stata v.17 (Stata Corporation, College Station, USA), and *R* v.4.3.1 with packages such as *tidyverse*, *lubridate*, and *zoo*. The variable that documented the patient’s occupation was condensed into eleven categories and the clinical signs and symptom variables were grouped into five categories. Demographic and clinical sign/symptom characteristics were abridged as proportions and frequencies to summarize the study data. Geographic data were downloaded in vector format from a public domain source ([www.naturalearthdata.com/downloads](http://www.naturalearthdata.com/downloads)). The *R* scripts used to summarize the data and construct figures will be made available upon reasonable request to the corresponding author.

**Results**

**
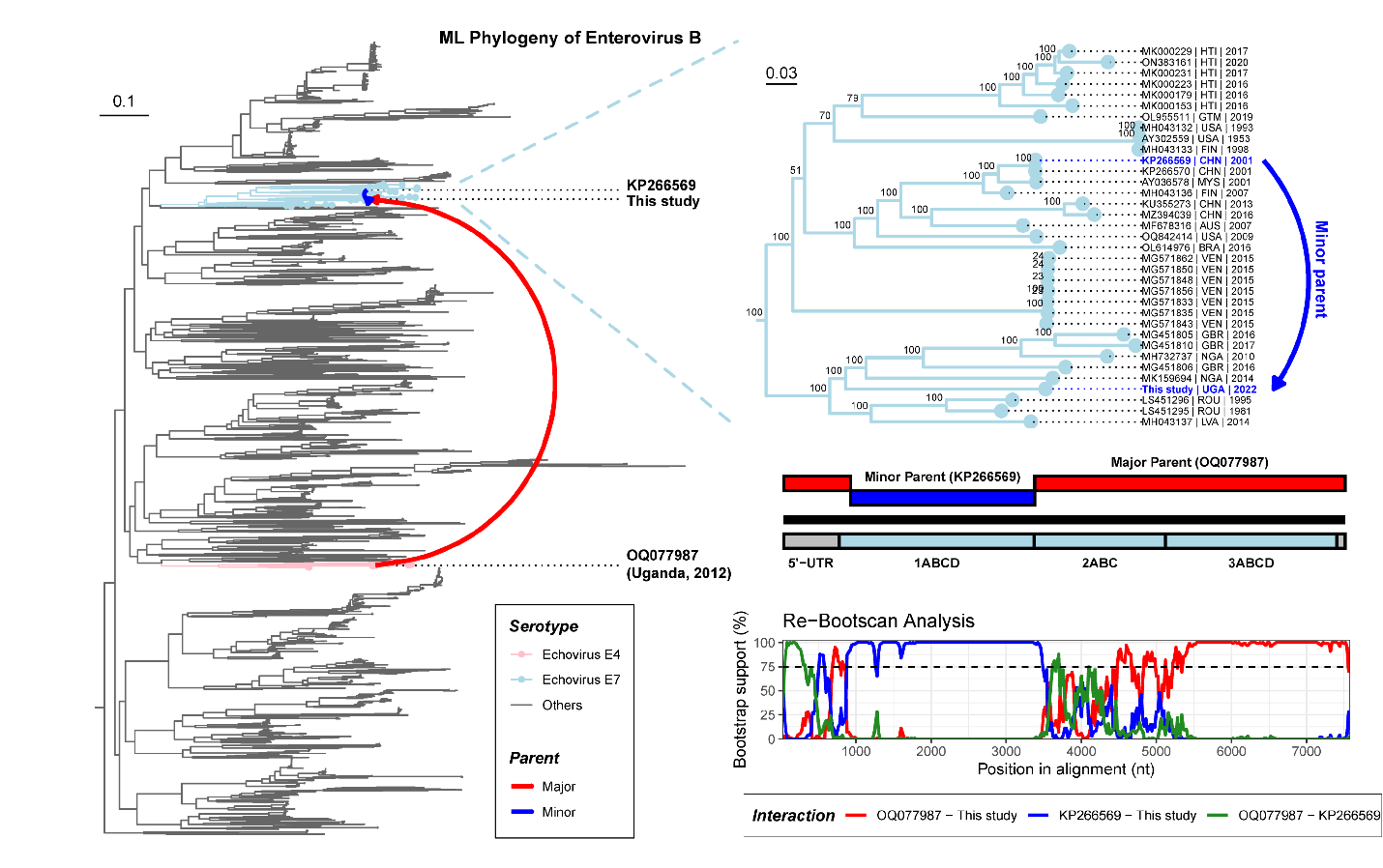
Supplemental Figure S1.** Maximum likelihood phylogeny of 1,766 complete Enterovirus B genomes (left and zoomed inset on top-right). An analysis of a reduced dataset of 343 genomes (bottom-right) identified a putative recombination event in the history of the consensus outbreak strain, with Bootscan analysis identifying the recombination breakpoints. The major and minor parents identified through the recombination analysis are denoted by arrows in the full-genome maximum likelihood tree.

**Supplemental Table S1.** (see Excel spreadsheet). Complete listing of metadata and NGS results for the n=134 specimens sequenced.

|  | 5’-UTR | 1ABCD | 2ABC | 3ABCD |
| --- | --- | --- | --- | --- |
| BLASTn Hit #1 | MH614923 \| EV-B80 (91%)  CHN \| 2011 | DQ227458 \| EchoV-7 (84%)  USA \| 2006 | LC321988 \| EchoV-9 (83%)  JPN \| 2017 | OQ077987 \| EchoV-4 (89%)  UGA \| 2012 |
| BLASTn Hit #2 | MH614924 \| EV-B80 (90%)  CHN \| 2011 | MH043136 \| EchoV-7 (84%)  FIN \| 2007 | KY981557 \| EchoV-11 (83%)  ISR \| 1997 | PP461527 \| CAV-B5 (89%)  NPL \| 2023 |
| BLASTn Hit #3 | MH614922 \| EV-B80 (90%)  CHN \| 2011 | LS451295 \| EchoV-7 (83%)  ROU \| 1981 | KY981560 \| EchoV-11 (83%)  ISR \| 1999 | MH933860 \| EV-B88 (88%)  CMR \| 2014 |
| BLASTn Hit #4 | JX476166 \| CAV-B3 (90%)  IND \| 2010 | MF678316 \| EchoV-7 (83%)  AUS \| 2007 | KY981559 \| EchoV-11 (83%)  ISR \| 1999 | MH685712 \| CAV-B4 (88%)  UGA \| 2013 |
| BLASTn Hit #5 | FJ460595 \| EchoV-7 (90%)  GRC \| 2010 | PP621607 \| EchoV-7 (83%)  NPL \| 2023 | HF948111 \| EchoV-25 (83%)  FRA \| 2005 | MG451806 \| EchoV-7 (87%)  GBR \| 2016 |
| BLASTn Hit #6 | MN817130 \| EchoV-11 (88%)  CHN \| 2019 | MT641377 \| EchoV-7 (83%)  GBR \| 2017 | FN691455 \| EchoV-6 (83%)  FRA \| 2000 | MF990292 \| EchoV-19 (87%)  ETH \| 2016 |
| BLASTn Hit #7 | KY981567 \| EchoV-7 (90%)  ISR \| 1999 | KU355273 \| EchoV-7 (83%)  CHN \| 2013 | MF838735 \| EchoV-3 (83%)  AUS \| 2006 | MF990293 \| EchoV-16 (86%)  ETH \| 2016 |
| BLASTn Hit #8 | EF634316 \| EchoV-11 (88%)  SVK \| 2008 | MZ394039 \| EchoV-7 (83%)  CHN \| 2016 | LS451295 \| EchoV-7 (83%)  ROU \| 1981 | KM201659 \| CAV-A9 (86%)  FRA \| 2013 |
| BLASTn Hit #9 | AJ577589 \| EchoV-11 (88%)  HUN \| 2004 | MN541041 \| EchoV-7 (83%)  CHN \| 2018 | MF990303 \| EchoV-19 (83%)  ETH \| 2016 | MF990301 \| EchoV-18 (86%)  ETH \| 2016 |
| BLASTn Hit #10 | MF521674 \| EV-B69 (91%)  NER \| 2015 | MT641401 \| EchoV-7 (83%)  GBR \| 2018 | KY981562 \| EchoV-11 (82%)  ISR \| 1999 | MH933854 \| EchoV-20 (86%)  CMR \| 2014 |

**Supplemental Table S2:** Top BLASTn results for each portion of the consensus outbreak EV-B genome found in this study. The nucleotide identity to the consensus outbreak genome is shown, as well as the collection country and year, for each accession. Abbreviations: EchoV – Echovirus; CAV – Coxsackievirus. Collection country follows the ISO 3166-1 alpha-3 code standard.

| *Recombination event* | | | *NT identity to parent in donated region* | | *Breakpoints (in sequence)* | |  |
| --- | --- | --- | --- | --- | --- | --- | --- |
| **Recombinant** | **Major Parent** | **Minor Parent** | **Major Parent** | **Minor Parent** | **Start** | **End** |  |
| Consensus | OQ077987 | KP266569 | 85.4% | 82.7% | 890 | 3327 |  |
|  |  |  |  |  |  |  |  |
| *Average p-values associated with seven detection methods* | | | | | | | |
| **RDP** | **GENECOV** | **Re-Bootscan** | **MaxChi** | **Chimaera** | **SisScan** | **3Seq** |  |
| 1.375 ×10^-35^ | 1.356 ×10^-28^ | 1.318 ×10^-30^ | 1.404 ×10^-25^ | 8.580 ×10^-30^ | 6.935 ×10^-40^ | 5.656 ×10^-50^ |  |

**Supplemental Table S3: Statistics from the recombination analysis of the consensus outbreak Enterovirus B genome recovered in this study.** Values are the direct output of the program RDP5.

**References**

[1] K. Katoh and D. M. Standley, "MAFFT Multiple Sequence Alignment Software Version 7: Improvements in Performance and Usability," *Molecular Biology and Evolution,* vol. 30, no. 4, pp. 772-780, 2013, doi: 10.1093/molbev/mst010.

[2] B. Q. Minh *et al.*, "IQ-TREE 2: New Models and Efficient Methods for Phylogenetic Inference in the Genomic Era," *Mol Biol Evol,* vol. 37, no. 5, pp. 1530-1534, May 1 2020, doi: 10.1093/molbev/msaa015.

[3] S. Kalyaanamoorthy, B. Q. Minh, T. K. F. Wong, A. Von Haeseler, and L. S. Jermiin, "ModelFinder: fast model selection for accurate phylogenetic estimates," *Nature Methods,* vol. 14, no. 6, pp. 587-589, 2017-06-01 2017, doi: 10.1038/nmeth.4285.

[4] D. F. Robinson, "Comparison of labeled trees with valency three," *Journal of Combinatorial Theory, Series B,* vol. 11, no. 2, pp. 105-119, 1971, doi: 10.1016/0095-8956(71)90020-7.

[5] G. W. Moore, M. Goodman, and J. Barnabas, "An iterative approach from the standpoint of the additive hypothesis to the dendrogram problem posed by molecular data sets," *J Theor Biol,* vol. 38, no. 3, pp. 423-57, Mar 1973, doi: 10.1016/0022-5193(73)90251-8.

[6] D. T. Hoang, O. Chernomor, A. von Haeseler, B. Q. Minh, and L. S. Vinh, "UFBoot2: Improving the Ultrafast Bootstrap Approximation," *Mol Biol Evol,* vol. 35, no. 2, pp. 518-522, Feb 1 2018, doi: 10.1093/molbev/msx281.

[7] D. H. Huson and C. Scornavacca, "Dendroscope 3: An Interactive Tool for Rooted Phylogenetic Trees and Networks," *Systematic Biology,* vol. 61, no. 6, pp. 1061-1067, 2012-12-01 2012, doi: 10.1093/sysbio/sys062.

[8] G. Yu, D. K. Smith, H. Zhu, Y. Guan, and T. T. Y. Lam, "ggtree: an r package for visualization and annotation of phylogenetic trees with their covariates and other associated data," *Methods in Ecology and Evolution,* vol. 8, no. 1, pp. 28-36, 2017-01-01 2017, doi: 10.1111/2041-210x.12628.

[9] A. Pikuła, A. Lisowska, A. Jasik, and L. J. Perez, "The Novel Genetic Background of Infectious Bursal Disease Virus Strains Emerging from the Action of Positive Selection," (in eng), *Viruses,* vol. 13, no. 3, Mar 2 2021, doi: 10.3390/v13030396.

[10] L. J. Perez, K. Forberg, G. A. Cloherty, and M. G. Berg, "Temporal and coevolutionary analyses reveal the events driving the emergence and circulation of human mamastroviruses," *Emerging Microbes & Infections,* vol. 12, no. 1, 2023, doi: 10.1080/22221751.2023.2217942.

[11] D. P. Martin *et al.*, "RDP5: a computer program for analyzing recombination in, and removing signals of recombination from, nucleotide sequence datasets," *Virus Evolution,* vol. 7, no. 1, p. veaa087, 2021, doi: 10.1093/ve/veaa087.
